# Vitrification of camel skin tissue for use as a resource for somatic cell nuclear transfer in *Camelus dromedarius*

**DOI:** 10.1007/s11626-021-00590-6

**Published:** 2021-05-20

**Authors:** Young-Bum Son, Yeon Ik Jeong, Yeon Woo Jeong, Xianfeng Yu, Lian Cai, Eun Ji Choi, Mohammad Shamim Hossein, Alex Tinson, Kuhad Kuldip Singh, Singh Rajesh, Al Shamsi Noura, Woo Suk Hwang

**Affiliations:** 1 UAE Biotech Research Center, 30310 Al Wathba, Abu Dhabi, United Arab Emirates; 2 Jilin Provincial Key Laboratory of Animal Model, College of Animal Science, Jilin University, Changchun, China; 3 Hilli E.T. Cloning and Surgical Centre Presidential Camels and Camel Racing Affairs, 17292 Al-Ain, United Arab Emirates

Animals with superior genetics could be reproduced by somatic cell nuclear transfer (SCNT). Generally, for SCNT viable cell lines are established from living animals (Jeong *et al*. 2020). However, complication arises when animals die suddenly due to a disease or accident (Jeong *et al.* 2013). A suitable tissue cryopreservation method that can preserve tissue integrity is important. The cryopreservation is a prominent method for preserving animal genetic resources, and autologous cells could be obtained from cryopreserved tissue which has numerous clinical applications (Buck *et al.* 1981; Taylor *et al.* 2019). Therefore, studies on tissue cryopreservation in several species, such as humans, rats, sheep, and dog, have been reported (Ishijima *et al.* 2006; Wang *et al.* 2008; Courbiere *et al.* 2009; Deng *et al.* 2009). However, studies have not been reported on the establishment of cells from cryopreserved tissues of *Camelus dromedariu*s (camel). In addition, limited studies have been reported on cloning through the SCNT technique using cells established from cryopreserved tissues without cryoprotectants (Hoshino *et al.* 2009; Zhang *et al.* 2012; Jeong *et al.* 2020).

Tissue cryopreservation is largely a slow-freezing and vitrification method. In general, programmed slow freezing is used for tissue freezing (Santos *et al.* 2007; Dalman *et al.* 2017; Lee *et al.* 2019). However, this technique requires long time for cryopreservation which could be detrimental (Santos *et al.* 2007; Wang *et al.* 2008; Dalman *et al.* 2017; Lee *et al.* 2019). Vitrification is a simple, inexpensive and rapid method for cryopreservation (Dalman *et al.* 2017; Lee *et al.* 2019; Santos *et al.* 2007; Wang *et al.* 2008). It could replace the slow freezing techniques as it prevents the formation of ice crystals due to an ultrarapid cooling procedure (Amorim *et al.* 2011). For vitrification of tissues, intracellular cryoprotectants such as dimethyl-sulfoxide (Me2SO), ethylene glycol (EG), and glycerol are mainly used (Santos *et al.* 2007; Amorim *et al.* 2011). Among these, EG has the lowest cytotoxicity and a rapid permeability across the cell membrane (Bautista and Kanagawa 1998; Eto *et al.* 2014; Kuleshova *et al.* 1999). Furthermore, EG leads to thinning of the phospholipid biolayers and diffuses through them (Hughes and Mancera 2014). Therefore, tissue vitrification using EG has been reported in various mammals (Isachenko *et al.* 2003; Yeoman *et al.* 2005; Gandolfi *et al.* 2006). However, studies on cryopreservation of tissue, except for ovarian tissue, are very limited, and no studies on tissue vitrification using EG in camels have been reported. Therefore, the present study established cell lines from vitrified camel skin tissues, their characteristics were evaluated accordingly, and the developmental efficiency of the embryo was analyzed after performing SCNT using these cells as donor cells.

All animal work was performed according to the animal study guidelines which were approved by the ethics committee at the Management of Scientific Centers and Presidential Camels (Accession No: PC4.1.5). To preserve the genetic resources of a camel (male) that died suddenly, vitrification was performed after biopsy of the ear skin tissue. As a control for evaluating the effectiveness of the tissue vitrification method, ear skin tissues were collected from 6 different camels (three males and three females). The chemicals used in this study were purchased from Sigma (St. Louis, MO) unless otherwise noted. We adjusted the pH to 7.4 and the osmolality to 280 mOsm/kg for all media.

**Figure 1.**
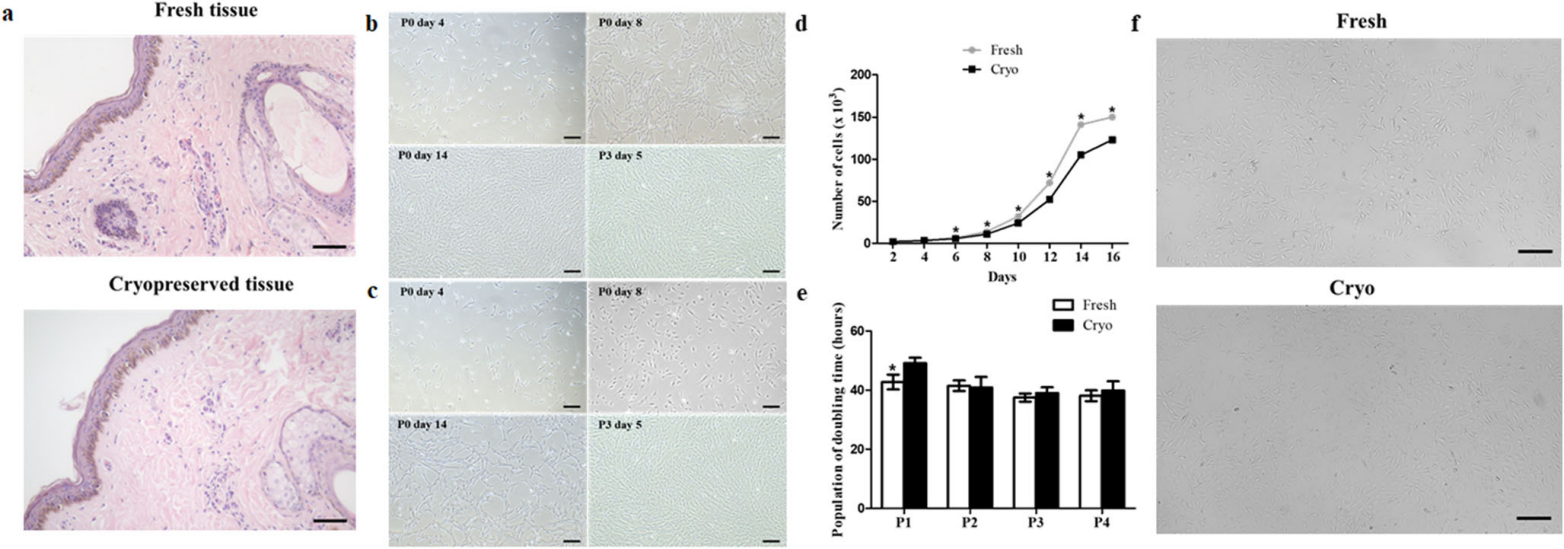
(*a*) H&E staining was conducted. Both tissues showed normal H&E staining. *Scale bar* = 800 μm. Morphology of camel fibroblasts from (*b*) fresh and (*c*) vitrified ear skin tissues (cryo), (*d*) cell proliferation analysis, and (*e*) population of doubling time assay (PDT). The fibroblasts from both groups showed a similar spindle-like morphology. The proliferative ability was decreased in the cryo group compared to the fresh group at the initiation of culture and at passage 1. However, there was no significant difference in the proliferative capacity between the two groups after passage 1. *Scale bar* = 200 μm. Data are represented by the mean ± SD of four independent experiments. *Asterisks* (*) indicate significant differences between groups (*P* < 0.05). (*f*) Cellular senescence was analyzed by SA-βgal staining. The number of SA-βgal positive cells was not different between the fresh- and the cryo groups. *Scale bar* = 200 μm.

We performed tissue vitrification as previously described with minor modifications (Santos *et al.* 2007). Briefly, tissues were washed with DPBS (Life Technologies, Carlsbad, CA) containing 10 μg/mL penicillin/streptomycin solution (Invitrogen). After that, tissues were cut into small pieces (1 cm^3^) at 25°C. The fragments were exposed to Dulbecco’s modified Eagle’s medium (DMEM; Thermo Fisher scientific, Waltham, MA) supplemented with 20% EG and 10% fetal bovine serum (FBS; Invitrogen) for 1 min and transferred to DMEM supplemented with 40% EG and 10% FBS using cryo-vials (Nunc, Roskilde, Denmark). The tissues were immediately transferred to liquid nitrogen (−196°C) and stored from 2010 to 2019. Both fresh and vitrified camel ear skin tissues were fixed with 10% formalin and embedded in paraffin. For histologic assessment, they were stained with hematoxylin and eosin (H&E) and the structure of ear skin tissues was identified as being in a normal state in both tissues (Fig. 1*a*).

The thawing of tissues was also conducted as previously reported with minor modifications (Santos *et al.* 2007). In brief, to remove the cryoprotectant during the thawing process, the vitrified tissues were kept at room temperature for 5 min and transferred to DMEM supplemented with 0.3 M sucrose and 10% FBS at 38°C for 5 min followed by DMEM supplemented with 0.15 M sucrose and 10% FBS for 5 min. After that, the tissues were washed 3 times using DMEM containing 10% FBS. We minced and treated with enzymatic dissociation of the vitrified and fresh tissues. After that, the cells were subsequently cultured in DMEM containing 10% FBS, 1% nonessential amino acids (Thermo Fisher Scientific, Waltham, MA), 1% antibiotic-antimycotic, and 0.1% β-mercaptoethanol (Thermo Fisher Scientific) at 38°C in a humidified atmosphere of 5% CO_2_. The number of viable cells and the cell proliferation capacity from vitrified and fresh tissues were analyzed as previously described with minor modifications (Legzdina *et al.* 2016; Shivakumar *et al.* 2016). Fibroblasts derived from both tissues were stained with 0.4% Trypan blue and the number of cells was counted every 2 d. Fibroblasts (2 × 10^3^) from both tissues were seeded into 6-well plates, and after every 72 h, the cells were counted using a hemocytometer. The population of doubling time (PDT) of the cells was calculated in accordance with the formula, PDT = log2 × T/(logNH – log NI), where T is the culture time, logNH is the harvest cell number, and log NI is the initial cell number. Camel fibroblasts from fresh and vitrified skin tissues showed homogenous plate-adherent and spindle-like cell morphology (Fig. 1*b*, *c*). In the initial culture process, fibroblasts derived from fresh tissues showed enhanced growth patterns compared with those derived from vitrified tissues (Fig. 1*d*). However, cell proliferation assessed by PDT showed similar values of proliferation after passage 1 (Fig. 1*e*). Therefore, we assumed that fibroblasts isolated from vitrified tissues could be used as donor cells in the SCNT process, and analyzed the characteristics of the cells.

**Figure 2.**
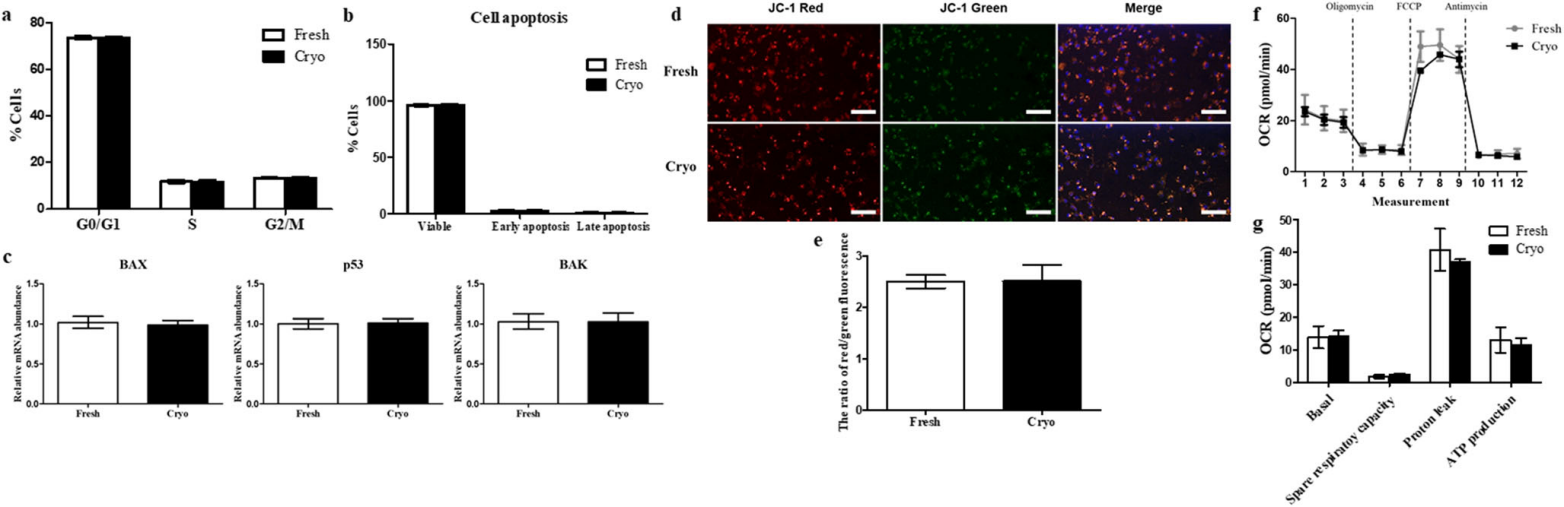
(*a*), (*b*) Analysis of the cell cycle and apoptosis in fibroblasts from fresh and vitrified ear skin tissues (cryo) at passage 3. No differences were observed between the fresh and cryo groups with respect to the portion of the cell cycle (*a*) and cellular apoptosis (*b*). (*c*) The mRNA expression levels of apoptosis-specific genes (Bax, Bak, and p53) were similar in both the fresh and cryo groups. Data are presented as the mean ± SD of four independent experiments. (*d*), (*e*) Analysis of mitochondrial membrane potential (ΔΨm) in fibroblasts from fresh and vitrified ear skin tissues (cryo). (*d*) Both types of fibroblasts were stained with JC-1 fluorescent dyes and DAPI. *Scale bar* = 100 μm. (*e*) The portion of red/green optical density was not significantly different between the fresh and cryo groups. Data are presented as the mean ± SD of four independent experiments. (*f*), (*g*) Oxygen consumption rate (OCR) assay of fibroblasts from fresh and vitrified ear skin tissues (cryo). (*f*) The cryo group showed similar mitochondrial respiration compared to the fresh group. (*g*) The values for basal respiration, proton leakage, spare respiratory capacity, and ATP production were similar in both groups. The *data point* in OCR represents the mean ± SD; *n* = 10 wells in independent experiments.

Cells lose their viability due to apoptosis induced during the tissue cryopreservation and thawing process (Amorim *et al.* 2011). This was one of the limitations of using tissue cryopreservation for securing adequate viable cells. Therefore, we cultured fibroblasts up to passage 3 for homogenization to use as donor cells for SCNT and analyzed cellular apoptosis that could have been induced by cryopreservation and the thawing of tissues. When cells from both fresh and vitrified tissues reached 80% confluence, senescence β-galactosidase (SA-βgal) was analyzed using the senescence β-galactosidase staining kit (Cell Signaling, Danvers, MA) following the manufacture’s protocol. The proportion of SA-βgal positive cells, an indicator of cellular senescence, was not different between the two groups (Fig. 1*f*) (Morgunova *et al.* 2016). To evaluate the conditions of the cells, we conducted cell cycle analysis following previous reports (Yang *et al.* 2006; Son *et al.* 2021). In brief, cells were fixed with 70% ethanol at 4°C overnight. After that, the cell pellet was washed with DPBS and treated with 100 μg/ml RNase A to ensure that only DNA was stained. Finally, we stained detached cells (1 × 10^6^) with propidium iodide solution (10 μg/ml), immediately analyzed them using flow cytometry (BD FACSVerseTM, BD Biosciences, Franklin Lakes, NJ), and categorized them into G0/G1, S, and G2/M phases. The rate of cell apoptosis was also evaluated using a Dead Cell FITC Annexin V Apoptosis Detection Kit (Invitrogen) according to the manufacturer’s instructions. The proportion of cells in the G0/G1 phase, which indicates cellular arrest, and S phase, the period in which cellular DNA is replicated, was not significantly different between the groups (Fig. 2*a*). Our results also showed that there was no significant difference in the portion of viable, early and late apoptosis between the two groups (Fig. 2*b*). Expression of apoptotic (BAX and p53), and anti-apoptotic (BAK) genes in fibroblasts derived from vitrified and fresh tissues was assessed using a real-time quantitative polymerase chain reaction (RT-qPCR). Total RNA was extracted from both cell lines at passage 3 from fresh and vitrified skin tissues using an easy-spin Total RNA Extraction Kit (Intron, Seongnam, Korea), and the synthesis of complementary DNA (cDNA) from total purified RNA (2 μg) with 10 μM oilgo(dT) primer at 42°C for 1 h using HisenScript RT PreMix kit (Intron). We performed qRT-PCR using a Rotor-Gene Q cycler (Qiagen, Hilden, Germany) and RealMOD^TM^ Green AP 5x qPCR mix (Intron) containing 100 ng cDNA and specific primer sets (Table 1). The RT-qPCR setting included initial activation at 95°C for 12 min, followed by 95°C for 15 s, 60°C for 25 s, and 72°C for 25 s. The expression of the gene was normalized to the mRNA levels of a control gene, glyceraldehyde 3-phosphate dehydrogenase (GAPDH). Pro-apoptotic-related (Bax, and p53) and anti-apoptotic-related (BAK) genes, assessed by RT-qPCR, were also not significantly different in these groups (Fig. 2*c*). These results showed that the vitrification of tissue method performed in this study did not significantly influence the expression level of pro-apoptotic and anti-apoptotic factors.

**Table 1.** List of primers used in the RT-qPCR analysis

Gene name (symbol)	Primers sequence	Product size (bp)	Anneal. Temp (°C)
BCL2-associated X protein (BAX)	F: CTGAGCAGATCATGAAGAC R: TACTGTCGAGTTCATCTCC	171	60
BCL-2 antagonist/killer 1 (BAK)	F: TACGACTCAGAGTTCCAG R: GCTGGTAGACATGTAGGG	169	60
Tumor protein 53 (p53)	F: CCATCTACAAGAAGTCAGAG R: AGTGGATAGTGGTACAGTCA	222	60
Glyceraldehyde 3-phosphate dehydrogenase (GAPDH)	F: GCTGAGTACGTTGTGGAGTC R: TCACGCCCATCACAAACATG	133	60

During the freezing and thawing process, cells are exposed to various physical and chemical stresses and damaged by reactive oxygen species (ROS) (Len *et al.* 2019). These ROS affect the cellular membrane and mitochondria and influence ATP synthesis through oxidative phosphorylation (OXPHOS) in the mitochondria of eukaryotes (Len *et al.* 2019). Therefore, we analyzed the mitochondrial membrane potential and oxygen consumption rate (OCR), an indicator of mitochondrial respiration in fibroblasts. We stained cells with JC-1 to evaluate the difference in mitochondrial membrane potential of the fibroblasts derived from fresh and vitrified tissues using a JC-1 mitochondrial membrane potential assay kit (Abnova, Taipei, Taiwan). After that, we analyzed the values of red-fluorescence aggregation, which indicates increased mitochondrial membrane potential, and green-fluorescence monomers, indicating decreased mitochondrial membrane potential (Fig. 2*d*). The ratio of red/green fluorescence histograms showed no significant difference between these groups (Fig. 2*e*). To confirm cellular respiration and energetics in fibroblasts from fresh and vitrified tissues, we conducted oxygen consumption rate analysis using a Seahorse XFp Cell Mito Stress Test (Seahorse, Agilent Technologies, Santa Clara, CA) following the manufacture’s protocol. Both types of fibroblasts were seeded at 50,000 cells/well in a Seahorse XF24 plate and cultured for 24 h before the assay. XF24 media supplemented with 2 μM rotenone, 1 μM carbonyl cyanide-4 (trifluoromethoxy) phenylhydrazone (FCCP), and 1 μg/ml oligomycin was loaded into the accompanying cartridge. Treatment with the drugs in the medium occurred at the time points specified (Fig. 2*f*). The OCR was monitored using a Seahorse Bioscience XF24 Extracellular Flux Analyzer. Each cycle was performed as follows: mix for 3 min, delay for 2 min, and then measure for 3 min. Basal respiration was evaluated before oligomycin injection, and proton leakage was calculated after oligomycin injection. ATP production was measured through the difference between basal respiration and proton leakage, and after FCCP injection, spare respiratory capacity was calculated through the difference between maximal respiration and ATP production. The OCR value was dramatically decreased after inhibition of the F0 ATP complex by treatment with oligomycin. After that, the OCR value was increased after the addition of FCCP, indicating that mitochondrial respiration was uncoupled in both groups. Our results revealed that the basal OCR indicating oxidative phosphorylation (OXPHOS) was not different in these groups (Fig. 2*f*). Furthermore, the values of basal respiration, spare respiratory capacity, proton leakage, and ATP production were also not significantly different between the groups (Fig. 2*g*). Overall, our data demonstrated that cells from vitrified tissues showed normal mitochondrial function compared to cells from fresh tissues.

We used fibroblasts derived from fresh and vitrified tissues as donor cells for SCNT. A total of 580 in vitro matured oocytes (96 ovaries) and 978 in vivo matured oocytes (78 oocyte donors) aged between 4 and 7 yr weighting 400 to 450 kg were used to evaluate the effect of donor cells on embryonic development after SCNT. The donors were injected with 5000 IU PMSG (Ceva, Libourne, France) and 500 μg of closprostenol (Jurox, Rutherford, Australia) to stimulate the ovary as previously reported (Tinson *et al.* 2000). After that, the donors were injected with 100 μg gonadorelin acetate (Vetoquinol, Paris, France) 25 to 28 h before OPU was conducted. The oocytes were obtained by an Aloka Ultrasound Unit (Aloka) with a needle guide (Aloka, Tokyo, Japan) using 60-cm, 18-gauge lumen needle in the follicles. Ovaries were washed with 0.9% saline solution, and follicles were aspirated using an 18-gauge needle with a 10-ml disposable syringe. The homogenous cytoplasm enclosed by at least three layers of compact cumulus cells was collected and washed with DPBS containing 5 mg/ml bovine serum albumin (BSA; Thermo Fisher Scientific) and 1% antibiotic-antimycotic. For in vitro maturation (IVM), COCs were cultured at 38°C in 5% CO_2_ in a humidified incubator for 42 h with IVM medium (IVF Bioscience, Falmouth, UK). After that, we conducted SCNT by the techniques as previously reported with minor modifications using in vitro and in vivo matured oocytes (Kim *et al.* 2012). The cumulus cells were denuded from oocytes by gentle pipetting with 0.1% hyaluronidase. The denuded oocytes were stained with 5 μg/ml bisbenzimide for 3 min and enucleated by aspirating the first polar body. After enucleation, a single donor cell was microinjected into the perivitelline space of the oocytes. These oocyte couplets were fused in fusion media containing 0.26 M mannitol, 0.1 mM MgSO_4_, 0.5 mM HEPES, and 0.05% (w/v) BSA with two DC pulses of 1.8 kV/cm for 15 μsec using a BTX Electro Cell Manipulator (BTX Inc., San Diego, CA). The reconstructed embryos were treated with 5 μM ionomycin for 3 min and with 2.0 mM 6-dimethylaminopurine (6-DMAP) in BO-IVC (IVF Bioscience, Falmouth, UK) in a humidified incubator with 5% CO_2_ at 39°C for 4 h. After activation, we cultured the embryos in 6 to 8 oil-covered droplets at 38°C in a humidified incubator with 5% CO_2_ and 5% O_2_ for 7 d. Our results showed that the rates of fused oocytes and cleaved embryos were similar in the fresh and cryopreserved groups (Table 2). The efficiency of blastocyst formation was slightly increased in the fresh group compared to the cryopreserved groups, but there was no significant difference (Table 2). The efficiency of fusion, cleavage, and blastocyst formation showed similar patterns in both the in vitro and in vivo matured oocytes (Table 2). Furthermore, similar to previous studies, the in vivo matured oocyte group showed higher cleavage and blastocyst formation rates than the in vitro matured oocyte group (Wani *et al.* 2018). Therefore, we confirmed that the source of oocytes was not related to the effects of the donor cells.

**Table 2.** Effect of donor cells on the fusion rate and efficiency of embryo development of somatic cell nuclear transfer using in vitro and in vivo matured oocytes

Nuclear transfer					
Donor cells	Source of oocytes	No. of oocyte			
		Reconstructed oocytes	Fused (%)	Cleaved (%)	Blastocyst (%)
Fresh	In vitro matured oocytes	301	213 (72.13 ± 3.86)	136 (65.83 ± 5.60)^a^	43 (22.55 ± 2.83)^a^
Cryo		279	196 (71.38 ± 1.86)	122 (62.47 ± 2.89)^a^	41 (20.69 ± 1.16)^a^
Fresh	In vivo matured oocytes	507	385 (75.85 ± 1.32)	285 (76.93 ± 1.98)^b^	191 (45.89 ± 1.85)^b^
Cryo		471	348 (75.79 ± 2.26)	292 (74.91 ± 3.13)^b^	183 (44.97 ± 3.05)^b^

Data are represented by the mean ± SE of four independent experiments

Lettered subscripts indicate statistical differences between groups (*P* < 0.05)

In conclusion, fibroblasts from vitrified tissues showed decreased proliferation compared with cells from fresh tissues in the initial culture process but showed similar patterns after passaging. Furthermore, our data showed similar values of mitochondrial metabolism, and the expression patterns of the cell cycle and apoptosis were also similarly observed after homogenization. Therefore, we determined that cells from vitrified tissues were suitable donor cells for SCNT, and confirmed that the efficiency of embryo development was similar to that of the fresh group. Taken together, camel SCNT embryos using cells from vitrified tissues were successfully developed to the blastocyst stage. These results suggested new approaches to tissue vitrification in preserving genetic resources.
